# Interobserver agreement of whole-body magnetic resonance imaging is superior to whole-body computed tomography for assessing disease burden in patients with multiple myeloma

**DOI:** 10.1007/s00330-019-06281-x

**Published:** 2019-07-02

**Authors:** Alta Y. T. Lai, Angela Riddell, Tara Barwick, Kevin Boyd, Andrea Rockall, Martin Kaiser, Dow-Mu Koh, Hind Saffar, Siraj Yusuf, Christina Messiou

**Affiliations:** 1 The Royal Marsden Hospital, London, UK; 2 Pamela Youde Nethersole Eastern Hospital, Chai Wan, Hong Kong SAR; 3 Imperial College Healthcare NHS Trust, London, UK; 4 Department of Surgery and Cancer, Imperial College London, London, UK; 5 The Institute of Cancer Research, London, UK; 6 Radiology Department, The Royal Marsden NHS Foundation Trust, London, UK

**Keywords:** Multiple myeloma, Diffusion magnetic resonance imaging, Whole-body imaging, Computed tomography

## Abstract

**Objectives:**

Whole-body MRI (WB-MRI) is recommended by the International Myeloma Working Group for all patients with asymptomatic myeloma and solitary plasmacytoma and by the UK NICE guidance for all patients with suspected myeloma. Some centres unable to offer WB-MRI offer low-dose whole-body CT (WB-CT). There are no studies comparing interobserver agreement and disease detection of contemporary WB-MRI (anatomical imaging and DWI) versus WB-CT. Our primary aim is to compare the interobserver agreement between WB-CT and WB-MRI in the diagnosis of myeloma.

**Methods:**

Consecutive patients with newly diagnosed myeloma imaged with WB-MRI and WB-CT were prospectively reviewed. For each body region and modality, two experienced and two junior radiologists scored disease burden with final scores by consensus. Intraclass correlation coefficients (ICC), median scores, Wilcoxon signed-rank test and Spearman’s correlation coefficients were calculated.

**Results:**

There was no significant difference in overall observer scores between WB-MRI and WB-CT (*p* = 0.87). For experienced observers, interobserver agreement for WB-MRI was superior to WB-CT overall and for each region, without overlap in whole-skeleton confidence intervals (ICC 0.98 versus 0.77, 95%CI 0.96–0.99 versus 0.45–0.91). For inexperienced observers, although there is a trend for a better interobserver score for the whole skeleton on WB-MRI (ICC 0.95, 95%CI 0.72–0.98) than on WB-CT (ICC 0.72, 95%CI 0.34–0.88), the confidence intervals overlap.

**Conclusions:**

WB-MRI offers excellent interobserver agreement which is superior to WB-CT for experienced observers. Although the overall burden was similar across both modalities, patients with lower disease burdens where MRI could be advantageous are not included in this series.

**Key Points:**

*• Whole-body MRI is recommended by the International Myeloma Working Group for patients with multiple myeloma and solitary plasmacytoma and by the NICE guidance for those with suspected multiple myeloma.*

*• Some centres unable to offer whole-body MRI (WB-MRI) offer low-dose whole-body CT (WB-CT).*

*• This prospective study demonstrates that contemporary WB-MRI (with anatomical sequences and DWI) provides better interobserver agreement in assessing myeloma disease burden for the whole skeleton and across any individual body region in myeloma patients when compared with low-dose whole-body CT.*

**Electronic supplementary material:**

The online version of this article (10.1007/s00330-019-06281-x) contains supplementary material, which is available to authorized users.

## Introduction

Radiographic skeletal survey (SS), which has been in widespread use for decades, only offers a very crude assessment of bone involvement in multiple myeloma. More recently, some centres have replaced skeletal survey with low-dose whole-body CT (WB-CT), which has been shown to have greater sensitivity [1–3]. However, because both skeletal survey and CT predominantly detect the destructive and/or reactive effects of myeloma disease on trabecular and cortical bone rather than disease within the bone marrow space, the sensitivity is inherently limited [4, 5]. The excellent soft tissue contrast of whole-body MRI (WB-MRI) allows direct imaging of the bone marrow, resulting in higher sensitivity and earlier detection. More recently, the superiority of WB-MRI over FDG PET-CT for disease detection has been demonstrated [6–8]. Consequently, the International Myeloma Working Group (IMWG) recommends WB-MRI for all patients with suspected solitary plasmacytoma or asymptomatic myeloma [9] and in the UK, the National Institute for Clinical Excellence [10] recommends WB-MRI for all patients with a suspected new diagnosis of myeloma.

A cost-effectiveness analysis of imaging strategies for myeloma diagnosis has reported that WB-CT and WB-MRI give the highest incremental net monetary benefit (INMB) under differing prevalence levels when compared with skeletal survey. Perhaps surprisingly, a negative INMB was reported for FDG PET-CT [11]. This suggests an approach using either WB-CT or WB-MRI could be cost-saving and health-improving. However, there is evidence that WB-CT has a lower lesion detection rate and understages patients compared with WB-MRI using conventional T1-weighted spin-echo and STIR MR sequences [5]. This margin of superiority is likely to be further improved with the addition of DWI, which is superior to conventional MRI sequences for detecting focal marrow lesions [12]. Nonetheless, as WB-CT is inexpensive and relatively quick to perform, many centres limited by MRI capacity may gravitate towards using non-contrast-enhanced WB-CT as standard imaging in this patient cohort.

However, sensitivity forms only part of the assessment of a diagnostic test. Diagnostic assessment tools for clinical or research applications must also be reliable. One of the measures of reliability is the interobserver agreement. To date, the interobserver agreement for WB-MRI and WB-CT is unknown in the context of disease detection in myeloma. Hence, the primary aim of this study is to compare the interobserver agreement and diagnostic sensitivity for disease detection of WB-MRI and WB-CT in patients with multiple myeloma.

## Materials and methods

### Study design and population

The study design was a prospective observational diagnostic test accuracy study, approved by the institutional review board. Patients with an established new diagnosis of myeloma as per IMWG criteria [9] who were planned to be imaged with both WB-CT and WB-MRI examinations before starting treatment between 2013 and 2017 were prospectively included following written informed consent. Patients with second malignancies were excluded.

### Image acquisition

Low-dose WB-CT (mean radiation dose 5 mSv) was acquired with a 128-slice CT scanner (Somatom Definition Flash, Siemens Healthineers), 120 kV, 50 mAs, and 0.5-s pitch. Axial images were reconstructed to 3 mm for review. All subjects were scanned supine with arms by their sides and the images were acquired from the skull vertex to the toes. No intravenous iodinated contrast was administered. Axial images of 1-mm slice thickness were reconstructed to secondary coronal and sagittal images for review. Axial images for bone and soft tissue assessment were reconstructed from the raw data obtained during scanning: for bone assessment using sharp (B50f) kernel and for soft tissue assessment using soft (B20f) kernel. Secondary coronal and sagittal reconstructions were generated using a slice thickness of 2 mm and slice increment of 1.5 mm. The typical duration for WB-CT examination was less than 5 min. The dose-length products (DLP) for the WB-CT examinations were recorded.

WB-MRI studies were performed using an Avanto 1.5-T system (Siemens Healthineers). All subjects were scanned supine with arms by their sides. Coil elements were positioned from the skull vertex to the knees. Sagittal T1-weighted images (TR 590 ms, TE 11 ms, FOV 400 mm, slice thickness 4 mm) and T2-weighted images (TR 2690 ms, TE 93 ms, FOV 400 mm, slice thickness 4 mm) of the spine were acquired, followed by axial diffusion-weighted sequences (single-shot double spin-echo echo-planar technique with STIR fat suppression in free breathing) using *b* values of 50 and 900 s/mm^2^ applied in 3 orthogonal directions and combined to the isotropic trace images. Diffusion-weighted images were acquired in multiple contiguous stations of 50 slices per station (slice thickness 5 mm, no gap, FOV 430 mm, phase direction AP, parallel imaging (GRAPPA) factor 2, TR 14800 ms, TE 66 ms, inversion time (TI) 180 ms, voxel size 2.9 mm × 2.9 mm × 5 mm, number of signal averages 4, matrix 150 × 150, bandwidth 1960 Hz per pixel). Axial T1-weighted Vibe Dixon 3D gradient echo breath-hold sequences (52 slices per slab, FOV 470 mm, TR/TE 7/2.38, 4.76 ms, flip angle 30, matrix 192 × 192) were also acquired, matching the acquisition stacks and partition thickness to the DWI. No intravenous gadolinium contrast was used. The typical duration for WB-MRI examination was 45 min.

### Image analysis

For each body region (skull, cervical spine, thoracic spine, lumbar spine, pelvis, ribs/other, long bones), two radiologists each with > 10 years of experience, blinded to clinical information and the MRI findings, made a categorisation of disease burden on WB-CT with a previously described scoring system [4, 13]. This allowed the assessment of the number of lesions (> 20, 10–20, < 10, 0) and largest lesion dimension (> 10, 5–10, < 5, 0 mm) for each body region, assigning a score from 3 to 0 for each characteristic (lesion number and size), i.e. score 3 for > 20 lesions, score 2 for 10–20 lesions, score 1 for < 10 lesions and score 0 for 0 lesions; score 3 for > 10 mm, score 2 for 5–10 mm, score 1 for < 5 mm and score 0 for 0 lesions. The maximum lesion dimension was measured on the window setting in which the lesion was the most readily appreciated. A total score was then calculated for the whole skeleton. To achieve the final observer scores, discrepancies were resolved by a consensus reading facilitated by a third experienced radiologist. At a different time, the image reading was repeated for the WB-MRI data with readers blinded to the clinical information and CT findings. The maximum lesion dimension was measured on the sequence in which the lesion was the most readily appreciated. The image reading for WB-CT and WB-MRI was subsequently repeated by another pair of junior radiologists (< 1-year experience as a consultant).

### Statistical analysis

Intraclass correlation coefficient (ICC) estimates and their 95% confidence intervals (95%CI) were calculated using a two-way random absolute single measures model. Statistical analyses were performed using IBM SPSS Statistics for Windows Version 25.0 (SPSS Inc.). ICC values less than 0.5 are considered to be indicative of poor reliability, values between 0.5 and 0.75 indicate moderate reliability, values between 0.75 and 0.9 indicate good reliability, and values greater than 0.90 indicate excellent reliability [14]. With two observers, to detect the smallest possible value of 0.5 for ICC, using a two-sided test, with a pre-specified 5% significance level test (*α* = 0.05) and a power of 80% (*β* = 0.2), the required sample size is approximately 22 [15]. The median and interquartile ranges (IQR) of the consensus observer scores on WB-MRI were compared with those on WB-CT, and the Wilcoxon signed-rank test was used to test the null hypothesis that the average signed rank of the two samples is zero. Spearman’s rank correlation coefficients were used to evaluate whether the WB-MRI and WB-CT scores of one observer correlated with the analogous scores of the other observer on a per-region and per-patient basis. A value of *p* < 0.05 was taken to be statistically significant in all tests.

## Results

A total of 22 patients with treatment-naïve symptomatic myeloma (mean age 61, range 36–72, 12 female and 10 male) were included. (Please see supplemental information for clinical details of the patient population.) A total of 154 body regions were scored for the presence of disease on CT and MRI. The interval between WB-CT and WB-MRI studies ranged from 0 to 26 days (mean 3 days, median 0 days). Mean bone marrow infiltration was 45% (range 15–80%). The mean DLP for WB-CT studies was 426.7 mGy/cm.

### Interobserver agreement

The first pair of experienced readers assessing WB-CT showed good interobserver agreement for scoring across the whole skeleton (ICC estimate 0.77, 95%CI 0.53–0.90). With regard to individual body regions, the agreement was best in the bony pelvis with an ICC estimate of 0.90 (95%CI 0.78–0.96), which is interpreted as good to excellent; however, agreement was poor to good in the rest of the body with ICC estimates ranging from 0.35 to 0.79 with wide 95% confidence intervals (Table 1). For WB-MRI, interobserver agreement (Table 2) was excellent in scoring for the whole skeleton (ICC estimate 0.98, 95%CI 0.96–0.99).

**Table 1:** Interobserver agreement as demonstrated by intraclass correlation coefficient (ICC) for scoring WB-CT for individual body regions and the whole skeleton

WB-CT	ICC between experienced observers (95% confidence interval)	ICC between junior observers (95% confidence interval)
Cervical spine	0.42 (0.04–0.70)	0.36 (scale not reliable–0.72)
Thoracic spine	0.46 (0.06–0.74)	0.30 (scale not reliable–0.70)
Lumbar spine	0.56 (0.16–0.80)	0.58 (scale not reliable–0.83)
Pelvis	0.90 (0.78–0.96)	0.83 (0.52–0.93)
Long bones	0.35 (scale not reliable–0.66)	0.63 (0.10–0.85)
Skull	0.41 (0.01–0.70)	0.52 (scale not reliable–0.80)
Rib and other bones	0.79 (0.56–0.91)	0.56 (scale not reliable–0.82)
Whole skeleton	0.77 (0.45–0.91)	0.72 (0.34–0.88)

**Table 2:** Interobserver agreement as demonstrated by ICC for scoring WB-MRI for individual body regions and the whole skeleton

WB-MRI	ICC between experienced observers (95% confidence interval)	ICC between junior observers (95% confidence interval)
Cervical spine	0.90 (0.79–0.96)	0.74 (0.38–0.89)
Thoracic spine	0.90 (0.78–0.96)	0.68 (0.25–0.86)
Lumbar spine	0.89 (0.75–0.95)	0.80 (0.53–0.92)
Pelvis	0.99 (0.97–0.99)	0.98 (0.95–0.99)
Long bones	0.92 (0.81–0.96)	0.89 (0.74–0.95)
Skull	0.89 (0.76–0.95)	0.72 (0.35–0.88)
Rib and other bones	0.92 (0.81–0.97)	0.91 (0.78–0.96)
Whole skeleton	0.98 (0.96–0.99)	0.95 (0.73–0.98)

**Fig. 1:**
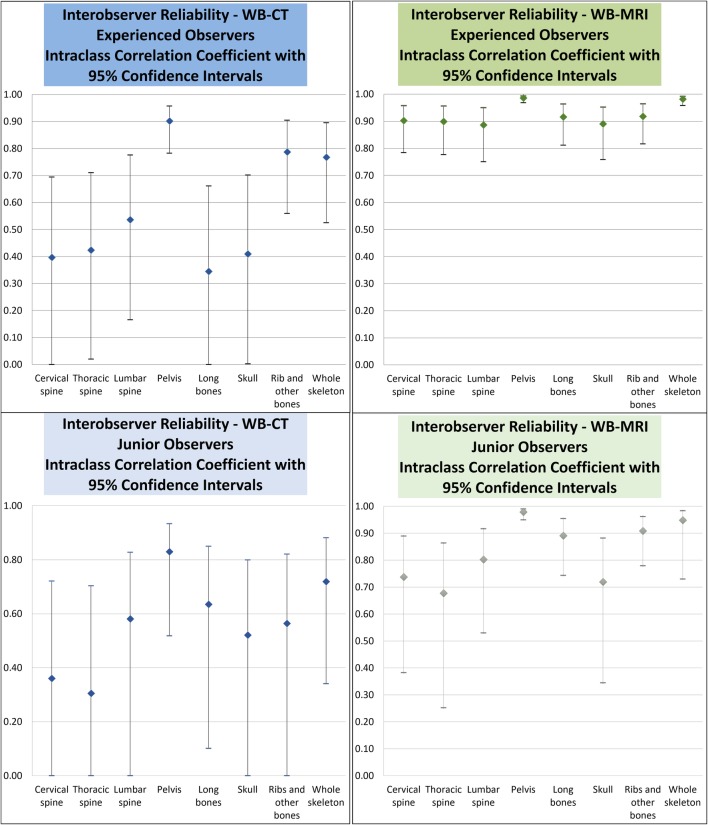
Interobserver agreement for WB-CT and WB-MRI expressed as intraclass correlation coefficient with 95% confidence intervals between a pair of experienced radiologists and between a pair of junior radiologists, respectively

Although WB-CT was found to be rather reliable in rating pelvic lesions, WB-MRI showed almost perfect reliability with robust 95% confidence intervals (ICC estimate of 0.99, 95%CI 0.97–0.99). WB-MRI also demonstrated good to excellent reliability across all other individual body regions with ICC estimates ranging from 0.90 to 0.92, all with narrow 95% confidence intervals (Table 2). ICC estimates and their 95% confidence intervals are illustrated in Fig. 1.

**Fig. 2:**
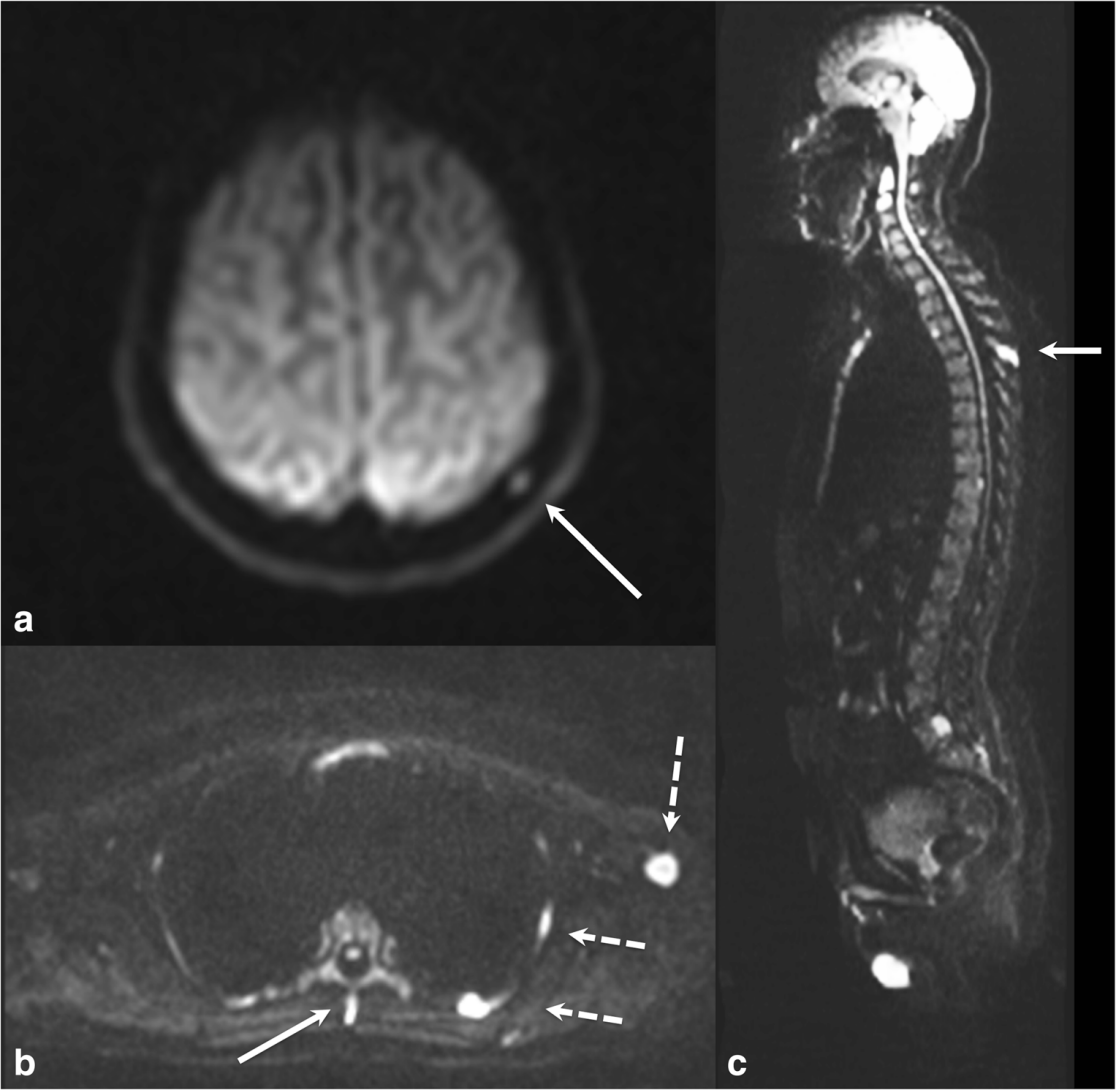
A 50-year-old gentleman with kappa light chain myeloma and 20% clonal cells on trephine bone marrow biopsy was found to have focal lesions in the cervical and lumbosacral spine, ribs and long bones on WB-MRI by all observers. The experienced observers detected additional small deposits in the left parietal skull vault (**a**) and in the spinous process of T4 vertebra (**b**, solid arrow) on an axial b900 DWI images; the latter being more evident on the b900 sagittal reformatted images (**c**, arrow). These subtle lesions were missed by the junior observers, resulting in a discrepancy in observer scores. The left humeral and left rib lesions (**b**, dotted arrows) were detected by all observers

Interobserver agreement between less experienced junior radiologists showed similar trends. For WB-CT, there was a moderate interobserver agreement for scoring across the whole skeleton (ICC estimate 0.72, 95%CI 0.34–0.88). With regard to individual body regions, the agreement was the best in the bony pelvis with an ICC estimate of 0.83 (95%CI 0.52–0.93), which is interpreted as moderate to excellent; however, agreement was poor to at most good in the rest of the body with ICC estimates ranging from 0.30 to 0.58 with wide 95% confidence intervals (Table 1). For WB-MRI, interobserver agreement (Table 2) was good in scoring for the whole skeleton (ICC estimate 0.95, 95%CI 0.73–0.98) (Fig. 2). For inexperienced observers, we should be cautious as although there is a trend for better ICC on whole skeleton WB-MRI, the confidence intervals do overlap.

### Observer scores for disease burden assessment

For all body regions and for the whole skeleton, median observer scores for WB-CT and WB-MRI are not significantly different from one another, as detailed in Table 3.

**Table 3:** Median and interquartile ranges (IQR) for consensus observer scores for WB-CT and WB-MRI. Wilcoxon signed-rank test (two-tailed) showed no statistically significant difference between the scores for WB-CT and WB-MRI

	WB-CT		CB-MRI			
	Median scores	IQR	Median scores	IQR	Z	*p* value
Cervical spine	0	0–2	0	0–0	− 1.51	0.13
Thoracic spine	0	0–2	0	0–2	− 0.36	0.72
Lumbar spine	0	0–2	0	0–2	− 0.32	0.75
Pelvis	1.5	0–4.25	1	0–4	− 0.39	0.70
Long bones	0	0–0	0	0–3	− 2.56	0.01
Skull	0	0–0.5	0	0–0	− 0.53	0.60
Rib and other bones	0	0–4	0	0–4	− 0.68	0.50
Whole skeleton	5	0–13	4	1.5–8.25	− 0.42	0.67

As listed in Table 4, there is a positive correlation between all scores rated by WB-CT and by WB-MRI, which is statistically significant in the lumbar spine, long bones, ribs and other bones and the whole skeleton. Correlation is highest for the lumbar spine (Spearman’s rho = 0.81, *p* < 0.01). Correlation is lowest and statistically insignificant for the thoracic spine (Spearman’s rho = 0.25, *p¸ = 0.56*). There is a moderate correlation for the whole skeleton (Spearman’s rho = 0.61) and the remaining individual body regions (Spearman’s rho = 0.33 to 0.61).

**Table 4:** Correlation between consensus WB-CT and WB-MRI scores for individual body regions and the whole skeleton, with *p* values for a two-tailed test of the null hypothesis rho = 0

Region	Correlation coefficient (Spearman’s rho)	*p* value
Cervical spine	0.36	0.10
Thoracic spine	0.25	0.56
Lumbar spine	0.81	< 0.01
Pelvis	0.39	0.71
Long bones	0.58	< 0.01
Skull	0.33	0.13
Rib and other bones	0.61	< 0.01
Whole skeleton	0.61	< 0.01

## Discussion

The study is the first to compare interobserver agreement between WB-MRI and WB-CT. Higher interobserver agreement of WB-MRI compared with WB-CT was demonstrated across the entire skeleton and for individual body regions by experienced and junior radiologists. Although higher scores were derived from WB-MRI compared with that from WB-CT for disease detection in long bones, there was no difference in the overall observer score. However, the study was limited by the inclusion of patients with confirmed myeloma and a high mean percentage marrow infiltration. The study therefore did not include a significant number of patients with lower disease burdens where MRI could be advantageous.

The clinical interpretation of whole-body cross-sectional studies is based on essentially visual assessment of the graphical representation of digital data, which is subject to variations due to observer experience, image interpretation and reading conditions. Hence, the interobserver agreement can be influenced by whether there is a distinct contrast between the normal and abnormal that can be easily and readily detected by the human eye. Inconsistency in interpretation is exacerbated in modalities where the difference between normal and abnormal is subtle, or when there is considerable overlap. As with any diagnostic test, to reach a valid diagnosis and judgement, it is desirable for the imaging used to assess myeloma to have a high degree of agreement. While agreement does not imply accuracy, a test with poor interobserver agreement cannot be reliably used in the clinical setting for the management decision-making [16].

**Fig. 3:**
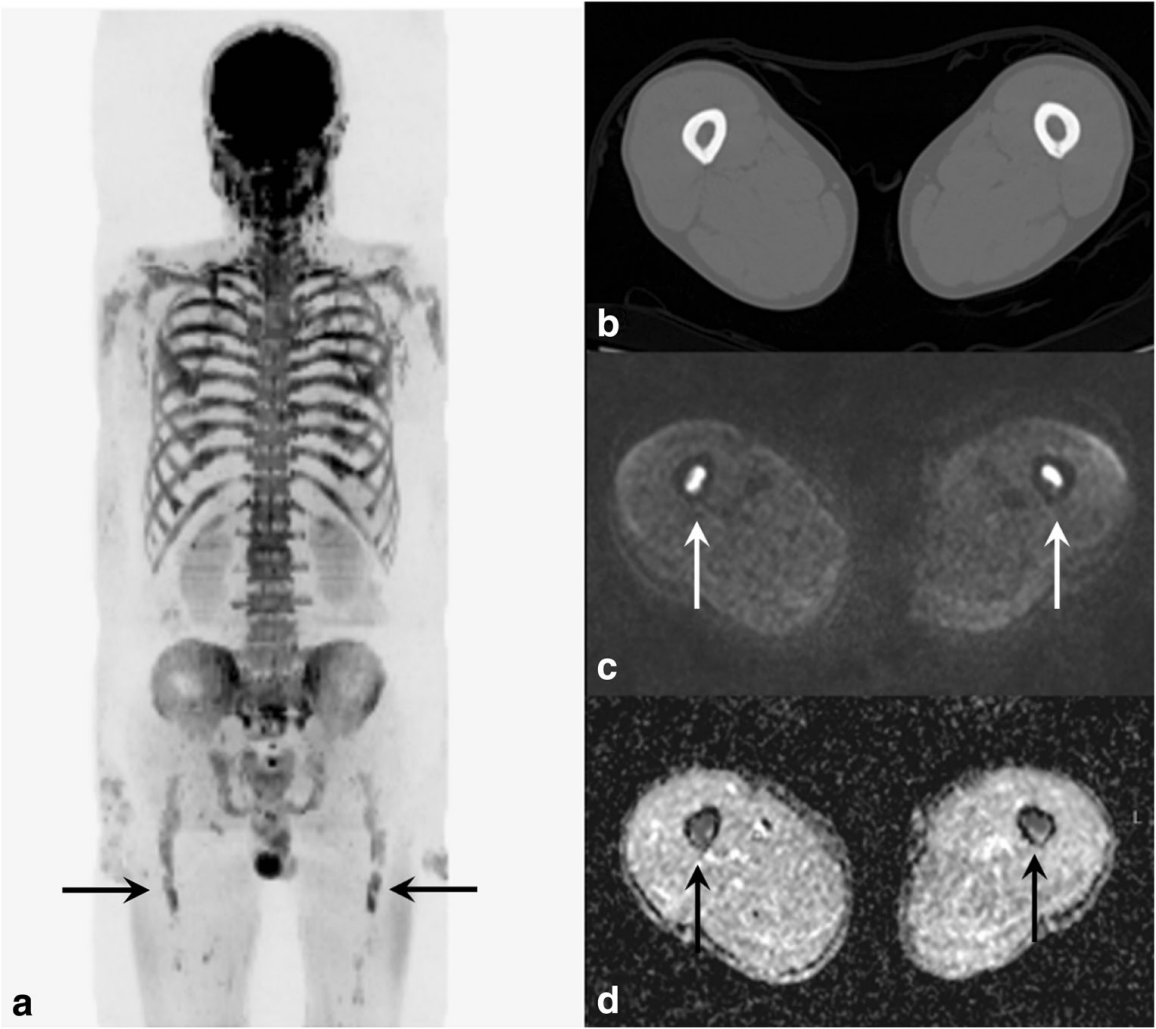
Marrow infiltration in bilateral femora in a 67-year-old male patient with IgG kappa myeloma and a high disease burden of 70% clonal cells on bone marrow biopsy. Disease is occult on axial CT (**b**) as there is no cortical destruction. However, widespread marrow disease is easily appreciable on WB-MRI maximum intensity projection (**a**, black arrows), *b* = 900 s/mm^2^ (**c**, arrows) axial images and ADC map (**d**, arrows), as regions of low signal intensities on the WB-DWI MIP image and as foci of high signal on the native b900 DWI image

DWI provides information on marrow cellularity, which has been shown to offer excellent visual contrast between normal and infiltrated hypercellular marrow compared with conventional MR sequences such as T1-weighted, short-tau inversion recovery and contrast-enhanced sequences [12, 17–19]. Moreover, CT has an inherent limitation in visualising the bone marrow and can often only reveal the secondary effects of myeloma on cortical and trabecular bone [5, 19], and the differentiation between normal bone marrow and lytic lesions is even more difficult in patients with generalised osteoporosis, which is often the case in myeloma patients, due to systemic factors [20] and age. The improved lesion conspicuity on MRI and in particular DWI, when compared with CT, can explain the higher interobserver agreement of WB-MRI, as illustrated in Fig. 3.

The challenges many centres face in providing WB-MRI services are predominantly capacity issues. Although WB-MRI has been shown to be well tolerated by patients with myeloma in a tertiary referral centre [7], acutely unwell patients may not be able to tolerate WB-MRI scans which can last for up to 45 min. Achieving faster scanning times is a priority for WB-MRI researchers but until that is achieved, shortened MRI protocols (i.e. spine and pelvis coverage only) and WB-CT are reasonable alternatives [21]. The position of WB-MRI is shifting from a state-of-the-art imaging technique to standard practice in oncological imaging, for disease detection, characterisation and therapy response in multiple myeloma.

## Conclusion

WB-MRI is an increasingly deployed imaging technique in cancer imaging. It can offer excellent interobserver agreement in quantifying disease burden for the whole skeleton and across any individual body region in myeloma patients. Future larger-scale multi-centre studies are anticipated to provide further evidence to support this practice.

## Electronic supplementary material

ESM 1 (DOCX 20 kb)

## Abbreviations

95%CI: 95% confidence intervals
DLP: Dose-length products
ICC: Intraclass correlation coefficients
IMWG: International Myeloma Working Group
INMB: Incremental net monetary benefit
IQR: Interquartile ranges
NICE: National Institute for Clinical Excellence
SS: Skeletal survey
WB-CT: Whole-body CT
WB-MRI: Whole-body MRI
