# Supplementary material for: Interobserver agreement of whole-body magnetic resonance imaging is superior to whole-body computed tomography for assessing disease burden in patients with multiple myeloma
Source: Eur Radiol. 2019 Jul 2;30(1):320–7. doi: 10.1007/s00330-019-06281-x (PMC6890623; doi:10.1007/s00330-019-06281-x)
Supplement: Supplementary file 1 — (DOCX 20 kb) [file 330_2019_6281_MOESM1_ESM.docx]

Additional information: Clinical details on patient population

| **No.** | **Type** | **Diagnostic features** | **Cytogenetics** | **High risk cytogenetic abnormalities** |
| --- | --- | --- | --- | --- |
| 1 | λ light chain | 10-20% affected cells on bone marrow trephine, light chain ratio | gain 1q +11 and -14/14q | High risk |
| 2 | IgG λ | 30-40% affected cells on bone marrow trephine, light chain ratio, renal impairment, anaemia, focal lesions on MRI | low level clonal loss at 17p13.1 | High risk |
| 3 | IgG κ | 60-65% affected cells on bone marrow trephine, light chain ratio, focal lesions on MRI | normal result | No |
| 4 | κ light chain | 20% affected cells on bone marrow trephine, light chain ratio, focal lesions on MRI | -14/14q | No |
| 5 | κ light chain | Chest wall plasmacytoma, light chain ratio, focal lesions on MRI | no cells on flow | N/A |
| 6 | κ light chain | Scapular plasmacytoma, light chain ratio, focal lesions on MRI | t(11;14)(q13;q32) | No |
| 7 | IgG κ | 70-80% affected cells on bone marrow trephine, focal lesions on MRI | Chromosome(11) +17 | No |
| 8 | Unknown | 10-15% affected cells on bone marrow trephine, light chain ratio, anaemia, focal lesions on MRI |  | N/A |
| 9 | κ light chain | 20-30% affected cells on bone marrow trephine, light chain ratio, focal lesions on MRI | 1q21.3, 14q32 | High risk |
| 10 | κ light chain | Ischial plasmacytoma, light chain ratio, anaemia, focal lesions on MRI |  | N/A |
| 11 | IgG κ | 60-70% affected cells on bone marrow trephine, light chain ratio, focal lesions on MRI |  | N/A |
| 12 | CD20 positive | Heavy infiltration affected cells on bone marrow trephine, light chain ratio |  | N/A |
| 13 | IgG κ | Positive bone marrow trephine, light chain ratio, focal lesions on MRI | no cells on flow | N/A |
| 14 | IgG λ | 60-70% affected cells on bone marrow trephine, other focal lesions on MRI | Normal | No |
| 15 | λ light chain | Vertebral plasmacytoma, light chain ratio, focal lesions on MRI | no cells on flow | N/A |
| 16 | IgG λ | 50% affected cells on bone marrow trephine, light chain ratio |  | N/A |
| 17 | IgG κ | 60% affected cells on bone marrow trephine, light chain ratio, focal lesions on MRI | Normal | No |
| 18 | λ light chain | 30-40% affected cells on bone marrow trephine, light chain ratio, focal lesions on MRI | t(4;14)(p16;q32) and (1q21.3) | Ultra high risk |
| 19 | Unknown | Sacral plasmacytoma, light chain ratio | t(14;16)(q32;q22) and 1q | Ultra high risk |
| 20 | κ light chain | 70-75% affected cells on bone marrow trephine, anaemia, other focal lesions on MRI | no cells on flow | N/A |
| 21 | IgG κ | 70% affected cells on bone marrow trephine, focal lesions on MRI | 1q21.3, -14/14q- and +11 | High risk |
| 22 | IgG κ | 70-80% affected cells on bone marrow trephine | -14/14q and 1q21.3 | High risk |
